# Absolute Memory for Tempo in Musicians and Non-Musicians

**DOI:** 10.1371/journal.pone.0163558

**Published:** 2016-10-19

**Authors:** Irene Gratton, Maria A. Brandimonte, Nicola Bruno

**Affiliations:** 1 Conservatorio di musica Giuseppe Tartini, Trieste, Italy; 2 Università Suor Orsola Benincasa, Napoli, Italy; 3 Università degli Studi di Parma, Parma, Italy; University of Western Ontario, CANADA

## Abstract

The ability to remember *tempo* (the perceived frequency of musical pulse) without external references may be defined, by analogy with the notion of absolute pitch, as *absolute tempo* (AT). Anecdotal reports and sparse empirical evidence suggest that at least some individuals possess AT. However, to our knowledge, no systematic assessments of AT have been performed using laboratory tasks comparable to those assessing absolute pitch. In the present study, we operationalize AT as the ability to identify and reproduce tempo in the absence of rhythmic or melodic frames of reference and assess these abilities in musically trained and untrained participants. We asked 15 *musicians* and 15 *non-musicians* to listen to a seven-step `tempo scale’ of metronome beats, each associated to a numerical label, and then to perform two memory tasks. In the first task, participants heard one of the tempi and attempted to report the correct label (*identification task*), in the second, they saw one label and attempted to tap the correct tempo (*production task*). A musical and visual excerpt was presented between successive trials as a distractor to prevent participants from using previous tempi as anchors. Thus, participants needed to encode tempo information with the corresponding label, store the information, and recall it to give the response. We found that more than half were able to perform above chance in at least one of the tasks, and that musical training differentiated between participants in identification, but not in production. These results suggest that AT is relatively wide-spread, relatively independent of musical training in tempo production, but further refined by training in tempo identification. We propose that at least in production, the underlying motor representations are related to *tactus*, a basic internal rhythmic period that may provide a body-based reference for encoding tempo.

## Introduction

The Italian word *tempo* (literally, ‘time’; plural: *tempi)* indicates the perceived frequency of the rhythmic pulse of music. Tempo reflects the frequency of beats, the “regularly recurring articulations in the flow of musical time” [1], which is measured by the ratio of beats over time (beats per minute or bpm, e.g. 120 bpm = 120 beats / 60 s = 2 Hz). Tempo is also identifiable by the time interval between beats (inter-onset-interval or IOI) that is the reciprocal of frequency expressed in milliseconds (e.g. 120 beats corresponds to IOI = 500 ms). In modern musical scores, tempo is shown as a metronome mark indicating the desired number of beats per minute with reference to a specific metrical level (e.g., quarter note = 100). Musical notation can also employ tempo indicators such as *Largo*, *Andante*, *Moderato*, *Allegro* or *Presto*. These Italian terms refer to a range of bpm, leading to some ambiguity and to some leeway for the interpreter's taste to choose the tempo of the performance. Finally, tempo should not be confused with time signature (also called ‘tempo’ in Italian). Time signatures, such as 4/4 or 6/8, are patterns of temporal organization, not frequencies, and indicate the *metre* of a piece. Metre reflects the structure of musical pulsations, organized into regularly recurring stronger and weaker pulses, in a hierarchy of beats levels [2]. Metre is intertwined with rhythm, which concerns patterns of durations and the organization of successive durations into coherent groups [3].

We can think of tempo in terms of mechanical series of metronome clicks, that is, as a sequence of isochronous time units. However, the connotation of tempo goes beyond the simple articulation from note to note. Tempo is necessary to achieve a sense of connectedness between successive musical events [3]. For this reason, it constitutes an essential element of musical expression, the “integrated bundle of musical elements to flow with a rightful sense”([4] p.99). Indeed, one of the more distinctive aspects of musical interpretation is the choice of tempo. The `correct’ tempo for any work may cover a rather wide range and be affected by many factors [3]. Before playing, a musician must be able to form some representation of the desired tempo. However, how this is done is not established [5]. In addition, during execution, performers must be able to control tempo globally [6], to keep the beat as a temporal pattern that conveys unity to the piece [7], and to return to the beat after the subtle deviations they adopt with specific expressive purposes (*expressive timing*, cf. Desain and Honing [8] and Clarke [9]).

Despite its importance in music, the psychological processes underlying memory for tempo are not well understood. It has been proposed that auditory features such as beat frequency and waveform frequency (the stimulus counterparts of tempo and pitch), may be stored in a memory system encoding sensory [10] instead of semantic properties [11]. However, such representations should be sufficiently abstract to support recognition of a piece despite transposition to a different register [12], change in instrumentation [13], and change in tempo [14]. At the same time, it seems reasonable to predict that such representations should preserve some sensory features [15, 16, 17, 18] given that, for instance, the appreciation of a performance requires the consideration of characteristics that are unique to that particular interpretation [19]. Thus, both sensory as well as more abstract features may be encoded in stored musical representations [11]. A key distinction here is that between absolute and relative encoding. In principle, to remember a piece we do not need the absolute durations of individual notes. People easily recognize songs even if the overall tempo has been changed, as long as relations between rhythmic elements remain constant. The same is true for pitch. We recognize a melody even if the key has been changed, provided that pitch relations defining the melody are preserved. This suggests that tempo, like pitch, may be most naturally encoded in relative rather than absolute terms. Relative codes represent structural relations within the components of a stimulus array, such as relations between individual notes within a melody. Absolute codes instead represent single attributes of specific stimuli, such as the pitch of a single note. Although relative codes are critical for pattern recognition, there is evidence that absolute representations can also be preserved in long-term memory. For instance, when individuals are asked to reproduce a familiar song, they tend to reproduce both the tempo and the pitch accurately [20]. This suggests that at least some individuals might be able to perceive and remember tempo absolutely [18]. Such an ability may be defined, by analogy with the notion of absolute pitch, as *the ability to identify or to produce a specific tempo without an external reference*, that is to say, *absolute tempo* (AT).

### Spontaneous tempo (*tactus*)

An extra-musical candidate as absolute reference for tempo might be the so-called *tactus*, the body-based reference rhythm for establishing the beat before metronomes [21, 3]. The Renaissance musical theorist Gaffurius (1496), for instance, equalled tactus to the pulse rate of a man breathing normally [22]. This idea resonates with more modern conceptions. Tactus as a hand movement to keep the time was first described in 1490 by Adam von Fulda [21] and recent studies emphasize kinaesthetic sensations in the connection between hearing rhythm and perceiving movement [9]. Tempo, as an expression of musical movement, recalls motion in physical space and alludes to physical motion of a body or limb. There is evidence that *final retard*, the expressive musical slowing at the end of piece or between sections of a piece, is interpreted relative to physical movement [23, 24, 25] and will tend to deviate from the preceding tempo according to specific rules [26]. Kronman and Sundberg [27] modelled final retard as a motion in constant negative acceleration, similarly to a runner slowing down. Thus, a framework for encoding tempo may be provided by constraints on actual human movement [28], not just by rhythmic physiological phenomena [29]. The basis for an internal beat reference may be constituted by typical rhythmic behaviours such as walking and running, which are by definition periodic. Interestingly, the mean stride for both adult men and women is about 117 steps per minute, men’s strides being longer than women’s strides, but not faster [28]. Although there is a great variability of this measure, the observed range (about 81 to 150 steps per minute) is very similar to the distribution of preferred tempi in a *finger-tapping* task [30, 28].

Tempo perception occurs in a specific range. When frequency is too high, individual beats merge into a continuous flow; when it is too low, they lose their temporal structure and are perceived as individual events. This range defines the *existence region* of tempo perception but cannot be defined exactly because transitions are gradual and individual differences are large. Parncutt [31] proposed 33 bpm as the lower limit and 300 bpm as the upper limit. London [32] set this range from about 30 bpm to 240 bpm. Other works report 24 bpm and 600 bpm [33]. We find similar limitations in tempo production. We cannot produce repetitive movements too fast, in a controlled manner, or too slow; in the latter case we loose the sense of continuity and feel a series of individual movements. The upper biomechanical limit rate for finger tapping is constrained by the maximum frequency at which the effector can move. According to some estimates, the upper limit is about 400 bpm [34] (see also [33]) and the lower rate limit is about 30 bpm [33]. These limits bear a certain degree of ambiguity, as continuation tapping is not strictly periodic, but exhibits longer-term fluctuations (for a review, see Large [35]). The production limits are therefore more precisely expressed as the limit IOIs (in this case, about 150 ms to 2 s). Tempi near the limits of the existence region are not easily perceived or produced. In contrast, an optimal range for tempo production and perception exists in the middle of this region. This *preferred tempo* region varies somewhat between individuals. On the average, the range has been estimated to be between 67 bpm and 150 bpm (see Moelants [36]) or approximately from 75 bpm to 200 bpm (see [33]). In this range, there is a peak of maximal salience, the so-called *spontaneous tempo*. Spontaneous tempo corresponds to a moderate frequency and has a special significance because we tend to gravitate towards it [37]. According to Parncutt [31], spontaneous tempo is around 100 bpm. Other authors have reported different values but all the reported frequencies are under 120 bpm [36]. McAuley [33] distinguished between spontaneous motor tempo (SMT), the natural or preferred rate of rhythmic motor activity (e.g., tapping), and preferred perceptual tempo (PPT) the rate of a series of sounds or lights that is judged to be neither too fast, nor too slow, but appears to be ‘just right’ [37, 38]. A representative value of SMT is 100 bpm (600 ms) but there are also large individual differences. SMT can vary from 300 bpm (200 ms) to 37.5 bpm (1,600 ms) [39, 38]. There are some evidences that young children prefer faster rates than old children and adults [39,38] and musicians and non-musicians often differ in their spontaneous rates [40]. The most commonly reported value for PPT is around 100 bpm, like SMT, but a wide range of values have also been reported over the years. Notably, SMT and PPT have comparable frequencies. Such correlation supports the view that motor and perceptual tempo preferences have a common psychological basis [38].

### Absolute tempo and the analogy with absolute pitch

We perceive pitch if a waveform frequency is between 16 and 20000 Hz [41]. Sounds of frequency less than 16 Hz are not ‘normally’ heard but may be felt bodily as vibrations [41]. Thus, both tempo and pitch are related to frequency. However, the analogy between pitch and tempo does not imply a spatial isomorphism [42]. A pitch relation (a melodic interval) refers to the distance between two pitches, measured on the degrees of the scale. In Western tonal music, pitches are organized such that a fixed pattern of inter-tone intervals, the diatonic scales, repeats at every octave in a cyclic structure [43]. In contrast, a tempo relation is not only a temporal distance, but it is also concerned with the velocity of motion between two onsets with respect to a metrical framework. Strong and weak beats organize in larger units over multiple time scales. These time scales constitute a hierarchy such that specific beats at each level periodically coincide [33]. A crucial aspect of this organization is again cyclicity: metre is a recurring pattern of time [44]. For this reason, the pitch-tempo analogy is better casted as a kind of cognitive isomorphism, based on a common cyclic structure that can be understood in terms of mathematical group theory [45] and described cross-culturally [46, 47].

Absolute pitch (AP) is the ability to recall pitch from long-term memory either to identify the pitch or the *chroma* (pitch class) of a tone presented in isolation, or to produce a specified pitch without an external reference [48, 49, 50]. AP does not involve supernormal perceptual mechanisms but is instead related to extremely well developed pitch memory and verbal labelling [51, 52, 53]. It is a rare ability that generally occurs in a small percentage of the general population, estimated to be no more than 0.01% (1 out of 10,000 [54, 48]) and it is strongly related to musical training [51, 49, 50]. AP is typically assessed by three kinds of tasks: Identification, production and memory decay. Possessors score well above chance on tests of these abilities [51]. Production and identification are highly correlated, although large individual differences exist. For example, not all individuals capable of absolute pitch identification are equally able at absolute pitch production [55]. Thus, these two abilities should be tested separately [52]. The phenomenon of AP provides strong evidence that at least some of us are capable of processing musically relevant representations without an external reference. While this is well established for pitch, however, whether a similar ability exists for tempo is much less clear.

It is well known that several great musicians, such as Mozart, Scrjabin, Messiaen, and Boulez, were AP possessors while others, such as Wagner, Čajkovskij, Ravel, or Stravinskij, were not [49]. Thus, absolute pitch is not necessary to become a musician; the basic skill exercised during musical training is *relative pitch*, the ability to recognize and produce pitch relations. Conversely, we have only anecdotal information on potential AT possessors. Bartók has been described as having an uncanny sense of tempo [56] and Toscanini was criticized for his ‘inexorable beat’ [57]. Reportedly, Ormandy was always able to produce exact tempo without a metronome. Italian pianist Vidusso was especially famous among his pupils for his tempo ability (personal communication). However, these anecdotal reports do not tell much about musicians who do not have this ability or on absolute tempo in non-musicians. Similarly to pitch, musical training stresses the role of tempo relations, such as for instance doubling or halving a tempo, and absolute tempo memory is typically not addressed [58]. Does AT exist? And if it does, is it relatively rare, like AP, or more common?

### Previous studies

In a seminal paper, Levitin and Cook [18] asked participants to name some of their favourite songs, checked that they knew them only in one canonical version, and recorded how they sang them. They found that participants reproduced tempo accurately: 72% of the productions were within ± 8% of the tempo of the known canonical version (*r* = .95). Productions showed minimal overestimation errors that could be explained by performance stress, which is known to induce speeding [59], by motor factors such as the tendency to perform faster rather than slower [60], or by perceptual factors such as the better perception of slowed-down in comparison to speeded-up performance [61]. These results suggest that tempo was encoded in absolute terms and could be retrieved when singing the songs, even by musically untrained participants. In a later study Pauws [62] requested trained and untrained singers to sing from memory melodies of familiar and less familiar Beatles songs, after listening to the original CD. Results supported the existence of absolute memory for tempo, irrespective of singing ability. Almost two thirds of the participants came reasonably close to the actual tempo on the CD, without differences between trained and untrained singers.

Lapidaki [63] investigated the consistency of tempo judgements, more specifically the consistency of ‘correct’ subjective tempo, over a period of time, during the listening process. Participants were asked, across four separate trials, to listen to the same six musical examples, from various musical styles, and to indicate whether the experimenter should set the tempo ‘faster’ or ‘slower’ until it sounded right to them. For a relatively small number of participants, the judgments were remarkably consistent across trials and relatively unaffected by such other factors as fatigue, mood, or time of the day. Given that participants were not allowed to have external references, such as a musical score or body movements, Lapidaki labelled this ability ‘absolute tempo’, by analogy with absolute pitch (see also [64]). However, we must consider that good performance may be biased by a strong memory for a small range of tempi, or by a subjectively preferred tempo that may vary in different contexts but remains mostly centred on 100 bpm (see above).

Collier and Collier [65, 56] studied jazz recordings in relation to the ability to double the tempo. They observed that when jazz musicians attempted to return to the original tempo after doubling, they did so with considerable accuracy [56]. The conclusion was that, given that the musicians were consistent across takes on different days, they had good tempo memory. These authors also stressed that jazz musicians seldom use metronomes, if ever, and that the possible use of metronomes to set initial tempi cannot account for the return to the original tempo. According to this memory hypothesis, authors suggest that musicians were relying on a sense of absolute tempo, analogous to absolute pitch [56]. Absolute tempo was displayed both in short-term memory, within each take, and in long-term memory, between takes. Finally, Fine and Bull [66] asked musicians and non-musicians to reproduce three tempi (35, 110 and 185 bpm) from memory by clapping. Results indicated that the slower and faster tempi were recalled better than the medium tempo, in accord with well-known serial position effects on free recall [67]. They did not find musical experience to affect tempo recall, but in their non-musicians group there were three participants with some musical experience and this could have diluted the difference between groups.

### The present study

Empirical studies indicate that the ability to remember tempo absolutely might exist. However, to our knowledge, no systematic assessments of absolute memory for tempo have been performed using laboratory tasks that could be compared to those used for assessing absolute pitch. In the present study, we sought to quantify the ability to identify or reproduce tempo in the absence of rhythmic or melodic frames of reference or external temporal anchors, in musically-trained and untrained participants. We asked participants to perform simplified identification and production tasks, which did not require musical training, and analysed accuracy and pattern of errors. To this aim, we developed a simple ‘tempo scale’ of metronome beats with artificial labels that were learned at the beginning of each testing block. To perform accurately on these tasks, participants needed to encode tempo information with the corresponding label, store the information, and recall it to give the responses. Our purpose was to test whether participants could memorize tempo without the musical cues provided by familiar songs or pieces used in previous studies. By using a simple sequence of beats, we completely eliminated melody and harmony cues, as well as some metric and rhythmic information (all the durations being the same), and focused on the specific and absolute components of tempo as beat frequency. Rhythmic information was not completely eliminated, as an isochronous series of beat remains a rhythmic frame of reference, but, indeed, it is a very minimal one.

## Methods

### Ethics statement

The research was conducted in compliance with the ethical standards of the Italian Board of Psychologists (see http://www.psy.it/codice_deontologico.html), the Ethical Code for Psychological Research of Italian Psychological Society (see http://www.aipass.org/node/26) and the Code of Ethical Principles for Medical Research Involving Human Subjects of the World Medical Association (Declaration of Helsinki). The experiment did not involve clinical tests or use of pharmaceuticals or medical equipment, did not require collecting health information from participants, and did not involve the use of deception or involve participant discomfort in any other way. For these reasons, and in accordance with its regulations, the approval of Ethics Committee for Clinical Research of the University of Trieste was deemed unnecessary.

All participants were 18 years or older at the time of the study. The study was conducted in established educational settings—the University of Trieste and the Trieste Music Conservatory—where students and colleagues are routinely involved in research activities as participants. All participants gave verbal consent after being adequately informed of the aims, methods, and procedure of the study. Potential participants were informed that their anonymity would be preserved at all stages. Verbal consent was a prerequisite for participating. The only information collected specifically for the purposes of this study were age and years of musical training. The names of those who gave verbal consent, namely the participants, were immediately transformed into coded identifiers (*Subject number*) and remained available to the first author only, who saved them in an encrypted file. Participants’ names never entered in any analyses of the data.

### Participants

Thirty volunteers participated in the study. Fifteen (nine women and six men) were undergraduate or graduate students of the University of Trieste (age range: 19–45 years, *M* = 26.9, *SD* = 7.2 years) with no specific musical training (‘non-musicians’). Fifteen (nine women and six men) were undergraduate or graduate piano students of the Trieste Music Conservatory (age range 18–47 years, *M* = 24.3, *SD* = 7.0 years) with at least 8 years (range 8–12 years, *M* = 10.2, *SD* = 1.2 years) of formal musical training (‘musicians’).

### Stimuli

The acoustic stimuli consisted of an ordered series of seven short sequences of metronomic clicks. We generated this series based on two criteria.

The first was that it could be reasonably assumed that the seven tempi were equally spaced perceptually. Based on well-established psychophysical principles, to achieve this we chose the target tempi to be at equal distances on a logarithmic scale and evaluated perceived differences based on assessments of tempo just-noticeable differences (JND) in the literature. Estimates of the JND in two-alternative forced-choice tempo perception tasks yield deviations from the actual tempo between 6.2% and 8.8% [68]. In continuation-tapping tasks, typical JNDs are between 7% and 11% from the correct tempo [69]. Listeners’ ability to detect tempo differences between 40 and 600 bpm for single interval sequences are approximately on the order of 6%. For multiple isochronous interval sequences, thresholds improve, on average, to 3%. Best performance, slightly below 2%, is found for sequences of 6 intervals of 400 ms, a 150 bpm tempo [68, 33].

The second criterion was that the ordering had to make sense from a musical point of view. Supporting this, we note that our tempo series can be considered a sort of tempo 'scale'. Although we acknowledge that the similarity should no be pushed too far, the tonal scale in the equal temperament system is precisely a series of equal logarithmic steps in frequency with one octave (1:2 frequency ratio) divided into 12 equal semitones [70]. We note further that the concept of a twelve-step logarithmic tempo series was employed by Karlheinz Stockhausen in his celebrated masterpiece *Gruppen for three orchestras* (1995–1957) as guide for the serial organization of the parts of the piece.

Based on these two criteria, we generated a temporal series of ‘semitempi’, starting at 40 bpm, by repeatedly multiplying by

$$\sqrt[{12}]{2}=1.059$$ (1)

which corresponds to increasing the frequency by 6% at each step. We obtained three 'octaves' of semitempi, the series (in bpm, rounded to integer):

40,42,45,48,50,53,57,60,63,67,71,76,80,85,90,95,101,107,113,120,127,135,143,151,160,170,180,190,202,214,226,240,254,269,285,302,320. (2)

From (2) we then chose seven bpm values, one every two steps (semitempo units), on the extension of one octave. This octave is roughly centred on 100 bpm and spans approximately the preferred tempo region as defined above. The seven bpm values (rounded to integer) were:

71–80–90–101–113–127–143bpm (3)

and correspond to the IOIs (defined above):

845.1–750.0–666.7–594.1–531.0–472.4–419.6ms

These bpm values are equally spaced on a logarithmic scale. We therefore assume that they are approximately equally spaced in psychological space (see for instance [71]). Furthermore, we can be reasonably sure from the above-mentioned estimates of tempo JND's that the tempi in (3), increasing in frequency by 12%, are perceptually distinguishable from one another.

For each bpm value in (3) we produced an MP3 audio clip with WireTape Studio, from an open source digital metronome [72] providing a clearly audible click. The timbre of the click closely resembled that of standard, commercially available metronomes. Each audio clip of metronomic clicks, henceforth simply `tempo’, lasted 10s. The number of beats in each tempo, rounded to integer, was 12, 13, 15, 17, 19, 21, 24. Participants were not told in advance that all tempi had the same duration. Thus, they had no reason to attempt to count the number of beats during the learning phase, a very hard task to accomplish accurately given the relatively small differences between these numbers and the difficulty of memorizing seven similar numbers. Finally, to provide verbal labels instead of hard-to-master metronomic designations in (3) we chose the numbers one to seven, one indicating the slowest tempo of the series and seven the fastest. To prevent participants from comparing tempi between trials and thereby use a relative rather than absolute code, between successive trials, we randomly presented a series of six 12s distractors consisting in musical and visual excerpts. These clips were extracted from the beginning of an abstract animated movie of the first movement, *Allegro*, of Bach’s cembalo Concert in F minor, BWV 1056. The full video and soundtrack are freely available online [73]. The mean tempo in all the excerpts was quarter note = 82 bpm.

### Procedure

The whole experiment was run on a MacBook Pro laptop computer using a PowerPoint slideshow. The experiment consisted of two tasks, *identification* and *production*. The completion of each task required about 10 minutes. Participants were tested individually in a silent room. Each participant completed the two tasks in two sessions separated by one to three days, depending on participants’ availability. At the beginning of each session, participants sat at the table in front of the laptop, and read the instructions for the specific task on the screen. The instructions were as follows (translated into English): "We will listen to seven sequences of metronome beats. They will be called ‘tempi’ and they will be ordered from slowest to fastest. Tempi will be identified by numbers from 1 (slowest) to 7 (fastest). In the test, you will be presented with a random sequence of these tempi (identification task version) / number (production task version). Your task will be, after each presentation, to report the number that in your opinion corresponds to the tempo you just heard (identification task version) / to tap on the table the tempo that corresponds to the presented number (production task version). In between presentations of tempi / number you will be presented with a brief audiovisual excerpt." After reading instructions, participants responded to two training items with tempi not included in the seven-tempi experimental scale. Afterwards, the ordered series of seven tempi on the screen, each lasting 10 s (learning set), was presented once, together with the image of the numerical label and with 4 s between each successive tempo (a black slide). We presented tempo from slowest to fastest in accord to the order of the Metronome series. Finally, a slide with the sentence: "Be ready as the test is about to start" was presented for 3 s and the test began. In the identification task, participants heard each randomly presented tempo (10s) and were required to identify it promptly, with a unique label, and to report verbally the corresponding number. In the production task, participants saw each numerical label randomly presented on the screen and were required to tap promptly, for 10s, with one finger on the table top to produce the corresponding tempo. After 10s, the end of the trial was signalled by the word ‘stop’ presented on the screen. After each response, the experimenter pressed the spacebar to continue. Participants heard the clicks through the computer internal speakers (they did not wear headphones). In each condition, participants performed seven trials; during the execution of tasks, they were not allowed to move any part of their body. All responses were recorded in MP3 format with a Yamaha POCKETRAK Recorder for later analyses.

### Design

We used a 2x2 mixed factorial design, consisting of two variables with two levels each: Training (musician vs. non-musicians) as a between-participants variable and task (identification and production) as a within-participants variable. The order of tasks was counterbalanced between participants. The independent variables were the level of expertise of participants and the experimental tasks. The dependent variables were the accuracy in retrieving the seven tempi as measured by the proportion of correct identifications and correct productions, as well as the errors as assessed by the distance between response and target tempo, expressed in number of semitempi, in the two tasks.

### Measures

Each participant’s productions recorded in MP3 format were imported in the open source software Audacity [74] to display sound amplitude vs. time, allowing us to clearly visualize the beat onsets. The produced tempo was computed by counting the number of beats in the time window defined by the onset of the second and second-last beats. The first and last beats in each series were excluded. Specifically, to obtain the mean produced tempo expressed in bpm we used

bpm=beats×60seconds (4)

In the identification task, the error was defined as the difference between the target and the response tempo, expressed in number of steps (semitempo units) on the scale described by (2). In this task, therefore, correct responses are simply responses that match the target labels. In the production task, conversely, the error was defined as the difference between the target and the response tempo, again expressed in semitempo units as the result of

$${\text{log}}_{\sqrt[{12}]{2}}\frac{response\;tempo}{target\;tempo}$$ (5)

such that, for instance, a 118 bpm response to the 101 bpm target corresponds to an error equal to 2.7 semitempi. We then considered as correct all responses falling within ± 1 semitempi from the target, corresponding to a bpm shift of ± 6%. We chose this range for several reasons. First, this range matches empirically observed precisions in tempo perception and production. Second, our chosen range corresponds to a bpm change of ± 6% and is a conservative estimate [75] that is adopted in most studies on absolute pitch where it corresponds to a resolution of one semitone [76, 77, 78]. Finally, given that the steps in scale (3) are divided by 2 semitempi intervals (a resolution of 12% between each contiguous step), our chosen range represents the smallest possible error in the identification task. This implies that this range allows the most meaningful comparison between accuracies in the two tasks.

## Results

### Raw responses in bpm units

**Fig 1:**
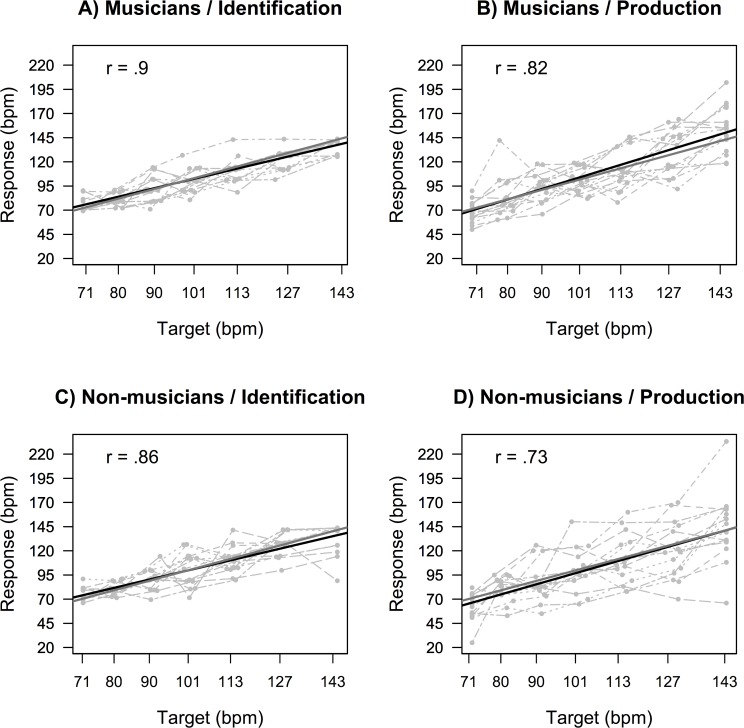
Response tempi as a function of target tempi. Response tempi as a function of target tempi expressed in bpm units, in each task and group. Each light grey point represents one response and each light grey connecting line identifies one participant. The dark grey solid line represents perfect accuracy (response = target). The black solid line is a linear regression fit to the group data.

Fig 1 presents scatterplots of response tempi as a function of target tempi, for each of the four conditions in Table 1. Bivariate distributions in the musicians and non-musicians groups were very similar between training groups (columns), whereas they differed clearly between tasks (rows). The bivariate distributions reveal two additional features characterizing this difference. First, the association between response and target tempi was slightly weaker in the production task (*r* = .82 and .73, for musicians and non-musicians, respectively) than in the identification task (*r* = .9 and .86). This feature is of limited interest as it is likely to reflect the different constraints on the response in the two tasks. For this reason, we will not discuss it further. Second, linear fits on the identification data indicated that in both conditions both training groups were reasonably accurate. Linear fits parameters on the identification data yielded slopes = 0.87 ± 0.04 and 0.87 ± 0.05 and intercepts = 12.60 ± 4.40 and 12.63 ± 5.42 for musicians and non-musicians, respectively. Similar fits on the production data yielded slopes = 1.13 ± 0.08 and 1.07 ± 0.10 and intercepts = -10.49 ± 8.23 and -9.79 ± 10.43. Thus, performance was always close to the expectation that average response tempo = target tempo for each target tempo value, although there was a slight tendency to underestimate in identification and a similar tendency to overestimate in production.

**Table 1:** Percentage of correct responses (standard deviations in parentheses) in the two tasks and groups.

	Identification	Production
**Musicians**	53.3 (3.5)	24.8 (5.7)
**Non—musicians**	43.8 (4.1)	19.0 (5.2)

### Number of correct responses

**Fig 2:**
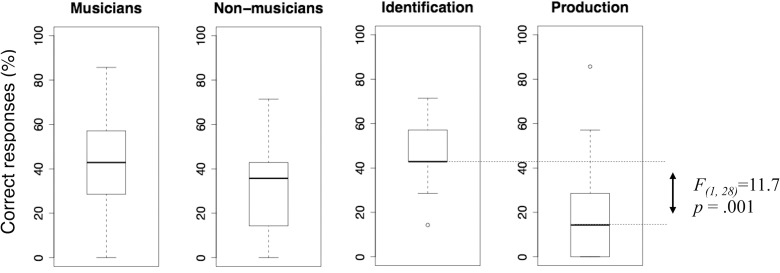
Distribution of percentages of correct responses. Box-plots summarizing marginal distributions of percentages of correct responses in the two groups and tasks. Top and bottom whiskers: max and min. Boxes: first and third percentile. Central horizontal line: median. The *F* test refers to the difference between the group means.

Table 1 presents percentages of correct responses by musicians and non-musicians in the two tasks. The corresponding marginal distributions are summarized by the box-plots in Fig 2. Raw data are included in Supporting Information file S1 Data. The distributions reveal substantial overlap between the two training groups, with the musicians’ median only slightly larger than that of non-musicians. Conversely, there is a clear difference between the two tasks. Given that the distributions were reasonably consistent with the assumption of multivariate normality, Shapiro-Wilk test *W* = 0.98, *p* = .53, and homogeneity of variance, Bartlett’s homoskedasticity test *χ*^*2*^_(1)_ = 0.12, *p* = .73, we subjected these data to a 2x2 mixed-model ANOVA with training (musicians, non-musicians) as the between-participants factor, task (identification, production) as the within-participants factor, and number of correct responses as the dependent variable. This analysis revealed a significant main effect of task, *F*_(1, 28)_ = 11.68, *p* = .001, *η*_*p*_^*2*^ = .37 whereas the main effects of training, *F*_(1, 28)_ = 2.76, *p* = .102, *η*_*p*_^*2*^ = .05 and the interaction, *F*_(1, 28)_ < 1, *η*_*p*_^*2*^ = .002, did not prove significant.

### Errors

Mean errors (difference between the response and the target tempo) and relative standard deviations are reported in Table 2. Note that errors are expressed in semitempo units, that is, unity corresponds to a 6% deviation relative to the target bpm and to roughly half the perceived difference between adjacent tempi in the graded series of our stimuli (assuming, as we have, that our series is approximately equally spaced psychologically, see Stimuli section). We observed that 48.6% of responses in identification and 22.0% in production fell within ± 1 semitempo from target and 87.6% of responses in identification and 47.1% in production fell within ± 2 semitempi (± 12%) from target. This is represented in Fig 1B and 1D), by the position of the data points relative to the marked areas that identify regions within one (light grey) and two semitempi (dark grey) from the line of perfect accuracy. Mean error magnitude is negative in each of the four conditions; this indicates a tendency to underestimate. Standard deviations are grater in non-musicians and in production.

**Table 2:** Mean errors. Mean errors expressed in number of semitempi from target. Standard deviations in parentheses.

	Identification	Production
**Musicians**	- 0.06 (0.18)	- 0.04 (0.27)
**Non—musicians**	- 0.11 (0.21)	- 0.97 (0.40)

### Comparison with chance performance

These results indicate that the pattern of responses was not random, but depended both on target tempo and on its ordinal position in the learning set. This in turn suggests that some participants were occasionally able to encode the presented tempo and retrieve it without a reference, that is, they might possess a form of absolute tempo. However, to determine how many participants may be assumed to possess this ability and to evaluate whether musical training modulates its prevalence, we need a criterion to identify participants who performed above chance. We defined this criterion as a threshold number *T* of correct responses, such that the probability *P* of achieving at least that number of correct responses is < 0.05.

Choosing *T* in the identification task is straightforward. The probability of a random correct response in a trial is 1/7. Using the binomial distribution, we can compute the vector of probabilities *P* of at least *k* random correct responses in 7 trials (see below). By inspecting these probabilities it appears that *T* = 4 satisfies the criterion.

          *k*     *P*

          0     1

          1     0.660083323

          2     0.263513866

          3     0.065229138

          4     0.010150047

          5     0.000970198

          6     0.000005221

          7     0.000000121

In the production task, chance level is lower because there are more than seven possible alternatives for each response; in this case, the choice of *T* is harder since there are several viable alternatives to calculate the probability of randomly producing a correct response. We compared two methods. In the first method, we computed repeated random permutations of the 210 participants’ productions, and assigned them as putative responses to the test. The number of correct responses after 100 permutation cycles was 2,600, corresponding to an estimated probability of a single correct random response *p* = .12. Using the binomial distribution, we find that the probability of 3 or more correct guesses is *P* = .042 whereas the probability of 2 or more guesses is *P* = .201. Hence, by this first method, we get *T* = 3. With the second method, we assumed that random responses are extracted from a uniform distribution of responses in a given range. We chose this range as the minimum and maximum bpm produced by all participants in all their responses, respectively, 23 and 233 bpm, corresponding to 40.1 semitempi units. In this case, the probability of giving the correct response by chance is estimated by the product of 2 probabilities *p*_*1*_ and *p*_*2*_, where *p*_*1*_ is the probability of producing a bpm in the range of correct responses, i.e. between 67 and 150.4 (respectively target 71 and 143) that corresponds to 14 semitempi units so that

$$p_{1}=\frac{semitempi\;btw\;67\;and\;150.4\;bpm}{semitempi\;btw\;23\;and\;233\;bpm}=\frac{14}{40.1}=0.35$$ (6)

and *p*_*2*_ is the probability that the bpm produced in this range is the correct response, or *p*_*2*_ = 1/7 because any bpm in this range is a potentially correct response. The composite probability of giving the correct response by chance is thus

$$p=p_{1}p_{2}=0.35\frac{1}{7}=0.05$$ (7)

in reasonable agreement with the estimated probability *p* = .12 calculated with the first method. Having calculated the probability of getting just one correct response by chance, using again with the binomial distribution we compute the probability of 2 or more guesses as *P* = .044 whereas the probability of 3 or more guesses as *P* = .0038. Thus, encouraged by the coincidence of results produced by both methods, we set the threshold for performance `above chance’ at *T* = 3 for the production task.

**Fig 3:**
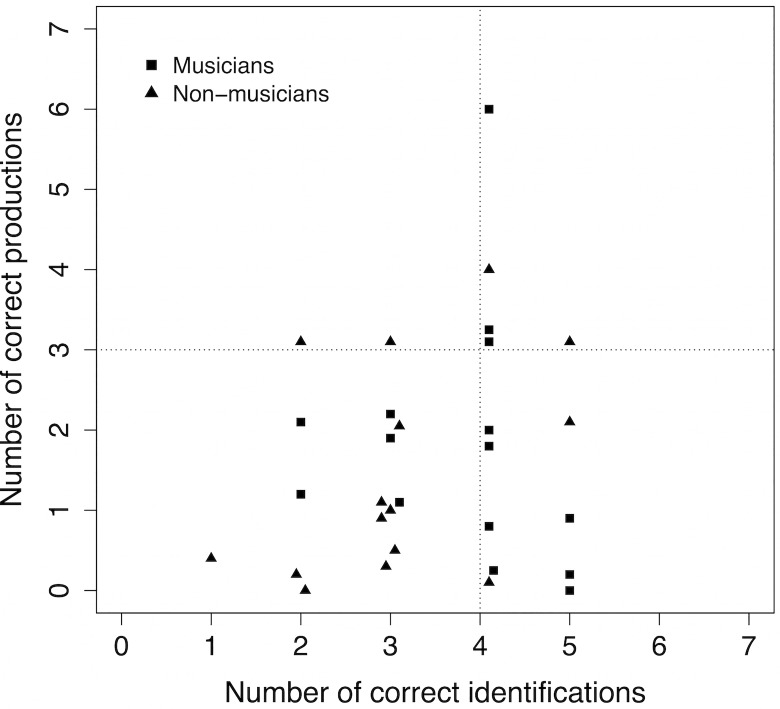
Number of correct responses for each participant in each task. Number of correct productions as a function of number of correct identifications in the musician and non-musician groups. Dotted lines identify criteria for above-chance performance. Each data point represents one participant. Some points are slightly displaced to avoid overlap with other points or the dotted lines.

Fig 3 plots the number of correct responses for each participant in each task. The dotted lines correspond to the chosen values of T and divide the graph in four quadrants: chance performance in both tasks (bottom left), above chance in both tasks (top right), chance performance in identification but above chance in production (top left), and chance performance in production but above chance in identification (bottom right). We can see that five participants (three musicians and two non-musicians) performed above chance in both tasks. Nine participants (seven musicians and two non-musicians) performed above chance in identification, but not in production. Two participants (both non-musicians) performed above chance in production, but not in identification. Thus, more than a half of the participants (53.3%) were able to perform above chance in at least one of the two tasks. The majority of these were musicians, whereas the majority of participants performing at chance in both tasks were non-musicians (nine out of fourteen).

**Fig 4:**
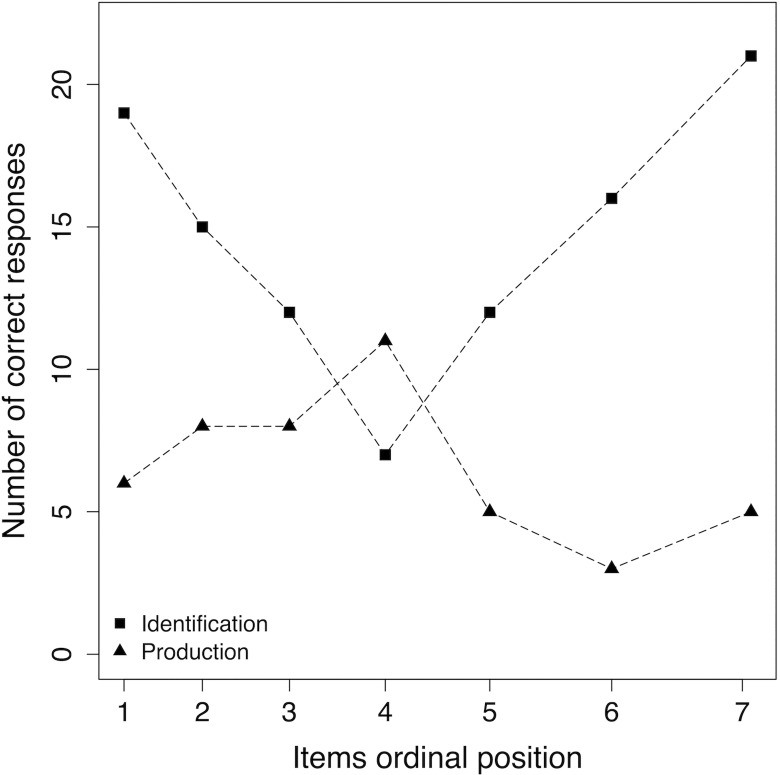
Average number of correct responses as a function of ordinal position in the learning set.

Finally, Fig 4 plots the average number of correct responses as a function of their ordinal position in the learning. The curves suggest that the two tasks were affected in dramatically different ways by ordinal position (An alternative possibility is that the tasks were affected by the items themselves. Although this seems unlikely, in principle it cannot be ruled out as the items were always presented in the same order during the learning phase). In identification, the curve was approximately U-shaped such that the initial and final tempi were identified best, whereas the central value (101 bpm) was the hardest. Out of 30 participants, only 7 (23%) correctly identified the central tempo; whereas these frequencies increased to 19, 15, 12, 12, 16, and 21 in the other six tempi (in order from 71 to 142, skipping 101bpm). A chi-square test of independence comparing frequencies of correct and incorrect responses within the central and all tempi revealed a significant association, *χ*^*2*^_(1)_ = 8.92, *p* = .003, *ϕ* = .21. In production, the curve was instead approximately an inverted U such that the central value was produced most accurately and the initial and final tempi less accurately. Out of 30 participants, as many as 11 (37%) correctly produced the central tempo; whereas these frequencies decreased to 6, 8, 8, 5, 3, 5 in the other six tempi (in order from 71 to 142, skipping 101bpm). Again a chi-square test of independence revealed a significant association, *χ*^*2*^_(1)_ = 4.46, *p* = .035, *ϕ* = .15. Thus, the curves in Fig 4 revealed a dissociation between tempo identification and production when performance in these two tasks was evaluated as a function of item ordinal position. This finding may stem from a previously unreported difference in the memory encoding of tempo and in its later retrieval under the conditions of our identification and production tasks. We will return to our interpretation of the dissociation in the final discussion.

### A note on distractors

The mean tempo in the music excerpts used as distractors was quarter note = 82 bpm. This value is therefore very similar to that of the second experimental tempo. It is known that when a finger-tapping task is accompanied by a distractor sequence, participants unconsciously tend to synchronize with the distractor sequence [34]. Our participants however did not synchronize with the distractor’s tempo since there is no evidence in the data of improved performance on the second item, or of a shift of produced tempi toward 82 bpm.

## Discussion

These results provide evidence that some individuals have the ability to retrieve the temporal rate of an acoustic event without a reference (absolute tempo, AT). When compared with the estimated prevalence of absolute pitch (AP) found in the literature (about 0.01%, see [48–55]), the number of individuals that performed better than chance in our tasks may be taken as support to the hypothesis that AT might be more common than AP. Also, in contrast with AP, which is generally considered to be relatively rare and strongly related to musical training [48], our results may be interpreted as evidence that AT is present in both musicians and non-musicians, although there is some evidence that musical training improves performance on tempo identification. It should be noted however that no accepted criterion exists for categorizing individuals as possessing AT. In the present study, as a first step in this direction we proposed a criterion based on a certain definition of chance performance. The current interpretation could however change if a different and presumably better criterion will be defined in future work.

Although our tasks did not differentiate sharply between musicians’ and non-musicians’ accuracies, we found a clear difference in performance between the identification and production tasks. Musicians showed better performance in identification in comparison to production and to non-musicians. This is especially surprising given that Western modern music is grounded on tonality, the systematic arrangement of pitches toward a referential pitch class (the tonic), whereas there is not a stable system of tempi. Our results are consistent with those of Pauws [62], who found absolute memory for tempo, but not for pitch, independent of singing ability. Participants were generally more accurate in identification, as one would expect given the nature of the two tasks. In the current data, approximately one every two participants performed above chance in identification, whereas only one out of four did so in production. Interestingly, when comparing performance against chance predictions the two tasks were affected in different ways by musical training. In the identification task, almost all of musicians were able to perform above chance, whereas the proportion of non-musicians that did so was approximately the same as the corresponding proportion in the production task. In the production task, most participants failed to perform above chance and, among those who did, musicians and non-musicians were present in approximately equal proportions. Surprisingly, musicians did not necessarily perform better than non-musicians in production tasks. This suggests that the ability to perform above chance in production is not related to musical training.

Presumably, tempo production involves more ‘natural’ abilities than tempo identification, as these abilities seem related to aspects of music cognition that are innate or learned very early [2, 79] and to motor processes [80, 29]. Music is not associated with a fixed semantic system but is, by essence, perceptually driven [11]. Perceptual learning from incidental exposure to the music of a culture provides the listener with implicit musical knowledge (automatically applied and not always available to conscious thought) of the structural pattern of that music [81]. Music is generally regarded as a product of human culture but core musical abilities are rooted in biological mechanisms [82]. For instance, a core mechanism enables most humans, independent of musical training, to sing a melody, to move in time with music, and to feel emotions when hearing music [83]; learning and singing a popular song are basic tasks that most of us can readily accomplish [82]. Peretz and Coltheart [83] describe these core mechanisms as a system of modules dedicated to the analysis or processing of different aspects of music. A modular account of music processing implies some degree of domain-specific processing and innateness [84]. Data on memory for tempo in one-week old infants [85] and the ability of newborns to perceive the temporal regularity of beats [86] also provide support for such innate components. However, it is prudent to consider that more general perceptual mechanisms may also account for the perceptual foundation of music [84].

Although our error analysis revealed that participants were generally accurate (see association between response and target tempi), the distribution of errors is also instructive. If there were no absolute memory for tempo, we would expect errors to be uniformly distributed. In contrast, we observed a clustering near the correct tempo (zero error); participants mainly made small errors, on average less than one semitempo. Finally, we observed a general tendency to give slower responses; this result is not consistent with the overestimation of tempo found by Levitin and Cook [18].

### Performance on the central value of the learning set

Finally, we found that 101 bpm, the central value of the learning set, was the best-produced and worst-identified tempo (Fig 4). Both our identification and production tasks required the conversion of tempo / label into label / tempo representations, entailing a mapping of the ordered series of tempo onto an ordered series of names and vice-versa [80]. In the identification task, the response, a conversion of a stimulus (tempo) to a name, is a cognitive process, a selection / competition among many names that are placed on an ordinal scale. In the production task, the response, consisting in the conversion of a name in a produced tempo, is a process that generates a motor program. We suggest that these features of the two tasks are presumably the reason for the observed two-pronged effect on the central value.

#### Identification

Our results in the identification task show the characteristic *bow effect* (called also *edge* or *end effect*) observed in absolute identification tasks when accuracy, the proportion of correct response, is plotted as a function of the ordered set of stimuli [87, 88]. Performance on stimuli that are either at the beginning or at the end of the range is better than performance on stimuli towards the middle of the range. To our knowledge this is the first investigation that reports a bow effect in absolute identification tasks with tempo in the auditory domain.

Most existing models of absolute identification assume that the magnitude of the stimulus is compared with a long-term representation of the magnitude of each stimulus from the set or of particular anchor values [87]. For instance, in *Thurstonian models*, long-term absolute magnitude information is represented in the positioning of criteria along a perceptual continuum [89, 90, 91]. In *exemplar models*, long-term absolute magnitude is represented in the stored stimulus-magnitude, stimulus-label pairs [92, 93, 94]. In *connectionist models*, long-term absolute magnitude is represented in the mapping between stimulus and response nodes [88]. In *anchor models*, finally, long-term absolute magnitude is represented as the memory for anchors at the edge of the stimulus range [95, 96] (for the empirical literature cf., among others, Stewart, Brown & Chater [87]; Lacouture & Marley [88]). In contrast to these models, the relative *judgment model* (RJM) does not assume long-term representations of absolute magnitudes. Instead, it assumes that responses are generated by comparing the current stimulus to the previous one, in conjunction with feedback from the previous trial [87, 97]. Proponents of the RJM assume that limits in performance are not perceptual in nature but relate to the judgment and that judgments are relative to the previous stimulus, not absolute. According to the RJM, a primary explanation of the bow effect is that for the first and last stimuli the opportunity to make mistakes is restricted (responses can be wrong only in one direction, being respectively larger or smaller than the correct response) whereas for the stimulus on the middle of the range, wrong responses can be either smaller or larger than the correct one. This limited possibility of error causes the peaks at each end of the range. Thus, absolute models assume substantial knowledge of the complete set of stimuli; relative models require only partial knowledge.

The present study was not designed to distinguish between these two classes of models. Further work is needed therefore to investigate the observed, and unexpected, bow effect. One interesting possibility with this respect might be to track responses in blocks with and without feedback. When in absolute identification the feedback is omitted, as in our study, participants use their previous response as the best estimate of the correct answer against which to base a relative judgment [87]. If RJM holds, therefore, in blocks without feedback we would expect to see that error rates vary systematically as a function of the correctness of previous responses, whereas in blocks with feedback this effect should disappear.

#### Production

In the production task we did not observe the bow effect. On the contrary, the central value of the learning set was, over the group of participants, the best produced. This result is not consistent with Fine and Bull who found that the medium among three tempi (110 bpm) was reproduced significantly worse than the first and last tempo [66]. We suggest that, in the production task, motor information implicated in the response generation has a specific link with spontaneous tempo or tactus. Several neuroscience studies suggest that there is a link between auditory and motor systems in rhythm processing (for a review of cognitive neuroscience literature see [98]); the motor system is activated not only during beat production, but also during beat perception. An auditory-motor model of rhythm perception was proposed by Todd and Lee [99], who considered two temporal dependent components: the Time domain and the Frequency domain processes, carrying out temporal segmentation and periodicity analysis, respectively. A third source of tempo dependency is imposed by sensory-motor processes, a representation of dynamic properties of the motor system that is necessary to plan an action in advance. Sensory motor components operate as a filter on the perceived rhythm; we may describe them as two dynamic systems associated with two types of motion: spontaneous foot tapping, which has a natural period of about 100 bpm [37], and the natural body sway, which has a period of about 12 bpm [98, 99]. The periodicity that is the nearest to the foot-tapping resonance will be the one favoured to select the tactus [98, 99].

Given the strong relationship between musical and physical motion [98, 99, 27] we might conclude that what we observed in our results is not, presumably, a memory effect, but a consequence of sensory motor integration whereby the role of the body (motor system) affects the choice/production of tempo [29]. In the learning set the tempo nearest to the periodicity of spontaneous tempo was 110 bpm, the central value. This is a knowledge-free competence, not affected by musical training [100], and could be a reasonable explanation for why 110 bpm was the best-produced tempo and why in the production task musicians did not perform better than non-musicians.

An alternative interpretation, plausible though partially speculative, takes into account the nature of the inter-trial distractor audio-visual sequence at test and its compatibility with the requirements of the tasks. It is commonly accepted (e.g., [101, 102]) that music shares important features with spoken language. For instance, both language and music involve the production and the organization of perceptually discrete elements into hierarchically structured sequences in accordance with syntactic principles [103, 104]. In addition, both need precise sequential timing, with audition playing a central role. Lastly, musical tasks share features with tasks used in motor learning, such as those involving movements of the hands and fingers with no verbal component. “From a listener’s perspective, music is a complex structured sequence of sounds, but from a performer’s perspective, it is also a long, complex sequence of motor acts” ([101] p. 52). In our task, the distractor sequence was introduced to prevent participants from comparing tempi between trials. However, being auditory in nature, it may have differentially impacted on the identification and production tasks, which relied on auditory recognition and motor reproduction, respectively. Thus, in the identification task, the distractor sequence may have prevented auditory rehearsal of the tempos, inducing reliance on their distinctiveness. The finding that in identification we observed the typical U-shaped serial position curve (i.e., the slowest and the fastest tempi were recognized better) is consistent with previous studies documenting ordinal position effects in auditory memory (e.g., [105, 106]). However, this is the first investigation that reports such effects in memory for tempo and, most importantly, shows that the effects reverse when participants are required to reproduce the encoded tempos motorically. We speculate that the auditory distractor task did not suppress motor memory, leaving kinaesthesic information available. Using such information, participants may have implicitly rehearsed motor movements using the spontaneous tempo (about 100 bpm) as a reference. Using this central value in this fashion would cause the serial position curve to take an inverted U-shape. Though speculative, this interpretation calls for more specific manipulations of the conditions for encoding and retrieval in tempo memory tasks. An obvious comparison under this respect might involve comparing conditions whereby participants are explicitly encouraged to move their hand to encode the tempo with conditions whereby they perform a different movement. Other investigations might consider stimuli not centred on 100 bpm to evaluate whether the statistics of stimulus array, rather than an internal reference, may provide constraints on accuracy. Exploring these issues may open interesting avenues for future investigations of this phenomenon.

## Supporting Information

S1 Data Contains dataset from experiment. (XLSX)

## References

[1] London J. Pulse In: Sadie S editor. The New Grove Dictionary of Music and Musicians. London: Macmillan; 2001–2002; XX: 599.

[2] Lerdahl F, Jackendoff R. A generative theory of tonal music Cambridge MA: The MIT Press; 1983

[3] London J. Rhythm In: Sadie S editor. The New Grove Dictionary of Music and Musicians. London: Macmillan; 2001–2002; XXI: 277–309.

[4] Epstein D. Shaping Time: Music, the Brain, and Performance New York: Schirmer Books; 1995.

[5] Ceriani G. Il senso del ritmo Roma: Meltemi Editore; 2003.

[6] Desain P, Honing H. Does expressive timing in music performance scale proportionally with tempo? Psychological Research. 1994; 56(4): 258–292.

[7] Clynes M, editor. Music, mind and brain: the neuropsychology of music New York: Plenum Press; 1982.

[8] Desain P, Honing H. Tempo curves considered harmful. In Kramer JD editor. Time in contemporary musical thought. Contemporary Music Review. 1993; 7(2): 123–138.

[9] Clarke E F. Rhythm and timing in music In: Deutsch D editor. The Psychology of Music. New York: Academic Press; 1999 pp. 473–500.

[10] Schatcher DL, Wagner AD, Buckner RL. Memory systems of 1999 In Tulving E, Craik FIM editors. Oxford Handbook of Memory New York: Oxford University Press; 2000 pp. 627–643.

[11] Peretz I, Zatorre RJ. Brain Organization for Music Processing. Annual Review of Psychology. 2005; 56: 89–114. 15709930 10.1146/annurev.psych.56.091103.070225

[12] Dowling WJ, Fujitani DS. Contour, interval, and pitch recognition in memory for melodies. Journal of the Acoustical Society of America. 1971; 49: 524–31. 5541747 10.1121/1.1912382

[13] Radvansky GA, Flemming KJ, Simmons JA. Timbre reliance in non musicians’ memory for melodies. Music Perception. 1995; 13: 127–40.

[14] Warren RM, Gardner DA, Brubaker BS, Bashford JA. Melodic and non melodic sequences of tones: effects of duration on perception. Music Perception. 1991; 8: 277–90.

[15] Halpern AR. Perceived and imagined tempos of familiar songs. Music Perception. 1988; 6: 193–202.

[16] Halpern AR. Memory for the absolute pitch of familiar songs. Memory & Cognition. 1989; 17: 572–81. 2796742 10.3758/bf03197080

[17] Levitin DJ. Absolute memory for musical pitch: evidence from the production of learned melodies. Perception & Psychophysics. 1994; 56(4): 414–423. 7984397 10.3758/bf03206733

[18] Levitin DJ, Cook PR. Memory for musical tempo: additional evidence that auditory memory is absolute. Perception and Psychophysics. 1996; 58(58): 927–935. 8768187 10.3758/bf03205494

[19] Raffman D. Language, Music and Mind Cambridge MA: MIT Press; 1993.

[20] Levitin DJ. Absolute pitch: self-reference and human memory. International Journal of Computing Anticipatory Systems. 1999; 4: 255–266.

[21] Brown HM, Bockmaier C. Tactus. In: Sadie S editor The New Grove Dictionary of Music and Musicians. London: Macmillan; 2001–2002; XXIV: 917–918.

[22] Dahlhaus C. Zur Entstehung des modernen Taksystems im 17. Jahrhundert. Archiv für Musikwissenschaft. 1961; XVIII: 223–240.

[23] Friberg A, Sundberg J. Does music performance allude to locomotion? A model of final ritardandi derived from measurements of stopping runners. Journal of Acoustical Society of America. 1999; 105(3): 1469–1484.

[24] Honing H. Some comments on the relation between music and motion. Music Theory Online. 2003 3; 9(1). Available: http://www.mtosmt.org/issues/mto.03.9.1/mto.03.9.1.honing_frames.html Accessed 18 September 2015.

[25] Honing H. The final ritard on music, motion and kinematic models. Computer Music Journal. 2003; 27(3): 66–72.

[26] Sundberg J, Verrillo V. On the anatomy of the retard. A study of timing in music. Quarterly Progress and Status Report. 1977; 18(2–3): 044–057.

[27] Kronman U, Sundberg J. Is the musical ritard an allusion to physical motion? In: Gabrielsson A editor. Action and Perception in Rhythm and Music. Stockholm: Royal Swedish Academy of Music; 1987 pp. 57–68.

[28] London J. Hearing Rhythmic Gestures: Moving Bodies and Embodied Minds. Keynote Addresses at the First International Music and Gesture Conference Norwich, UK. 2003 August. Available: http://www.people.carleton.edu/~jlondon/Keynote%20Webdocument.htm Accessed 18 September 2015.

[29] Todd NM, Lee C, O’Boyle D. A sensorimotor theory of temporal tracking and beat induction. Psychological Research. 2002; 66(1): 26–39. 11963275 10.1007/s004260100071

[30] Whittle MW. Clinical gait analysis: A review. Human Movement Science. 1996; 15(3): 369–387.

[31] Parncutt R. A Perceptual Model of Pulse Salience and Metrical Accent in Musical Rhythms. Music Perception 1994 11(4): 409–464.

[32] London J. Hearing in time: Psychological aspects of musical meter Oxford: University Press; 2004.

[33] McAuley JD. Tempo and rhythm In Jones M R, Fay R R, Popper A N editors. *Music perception*: *Springer handbook of auditory research*. New York: Springer; 2010 pp. 165–199.

[34] Repp B H. Sensorymotor synchronization: A review of the tapping literature. Psychonomic Bulletin & Review. 2005; 12(6): 969–992. 16615317 10.3758/bf03206433

[35] Large EW. Resonating to musical rhythm: theory and experiment In Grondin S editor. The Psychology of Time; Bingley, UK: Emerald; 2008 pp. 189–213.

[36] Moelants D. Preferred tempo reconsidered. In: Stevens C, Burnahm D, Mc Pherson G, Schubert E, Renwick J editors. Proceedings of the 7th International Conference on Music Perception and Cognition; 2002 Sydney, Australia. Sydney: Casual Productions; 2002. pp. 580–583.

[37] Fraisse P. Rhythm and tempo In: Deutsch D editor. The Psychology of Music. New York: Academic Press; 1982 pp. 149–180.

[38] McAuley JD, Jones MR, Holub S, Johnston HM, Miller NS. The time of our lives: life span development of timing and event tracking. Journal of Experimantal Psychology: General. 2006; 135 (3): 348–367 10.1037/0096-3445.135.3.348 16846269

[39] Drake C, Jones MR, Baruch C. The development of rhythmic attending in auditory sequences: attunement, referent period, focal attending. Cognition. 2000; 77: 251–288. 11018511 10.1016/s0010-0277(00)00106-2

[40] Zamm A, Pfordresher P, Palmer C. Temporal coordination in joint music performance: Effects of endogenous rhythms and auditory feedback. Experimental Brain Research. 2014; 233 (2): 607–615 doi: 10.1007/s00221-014-4140-5 25399244 10.1007/s00221-014-4140-5 PMC4295031

[41] Moore BCJ. Hearing and psychoacoustic In: Sadie S editor. The New Grove Dictionary of Music and Musicians. London: Macmillan; 2001–2002; XX: 294–299.

[42] London J. Some non-isomorphisms between pitch and time. Journal of Music Theory. 2002; 46(1/2): 127–151.

[43] Krumhansl C. l. The Psychological Representation of Musical Pitch in a Tonal Context Cognitive Psychology. 1979; 11: 346–374

[44] London J. Hierarchical representation of complex meters. In 6th International Conference on Music, Perception and Cognition; Keele University, United Kingdom, August, 5–10, 2000.

[45] Pressing J. Cognitive isomorphisms between pitch and rhythm in world musics: West Africa, the Balkans and Western tonality. Studies in Music. 1983; 17: 38–61.

[46] Stevens C. Cross-cultural studies of musical pitch and time. Acoustical Science and Technology. 2004; 25(6): 433–438.

[47] Bar-Yosef A. A cross-cultural structural analogy between pitch and time organizations. Music Perception: An Interdisciplinary Journal. 2007; 24(3): 265–280.

[48] Ward WD. Absolute pitch In: Deutsch D editor. The Psychology of Music. New York: Academic Press; 1999 pp. 265–298.

[49] Parncutt R, Levitin DJ. Absolute pitch In: Sadie S editor. The New Grove Dictionary of Music and Musicians. London: Macmillan; 2001–2002; I: 37–39.

[50] Takeuchi AH, Hulse SH. Absolute-pitch judgments of black—and white key pitches. Music Perception. 1991; 9: 27–46.

[51] Levitin DJ, Rogers E. Absolute pitch: perception, coding and controversies. TRENDS in Cognitive Science. 2005 1; 9(1): 26–33. 10.1016/j.tics.2004.11.007 15639438

[52] Levitin DJ. Memory for musical attributes In Cook PR editor. Music, cognition and computerized sound: An introduction to psychoacoustics. Cambridge MA: The MIT Press; 1999.

[53] Massaro DW. Perceptual images, processing time, and perceptual units in auditory perception. Psychological Review. 1972; 79(2): 124–145. 5024158 10.1037/h0032264

[54] Profita J, Bidder TG. Perfect pitch. American Journal of Medical Genetics. 1988; 29: 763–771. 3400722 10.1002/ajmg.1320290405

[55] Takeuchi AH, Hulse SH. Absolute pitch. Psychological Bulletin. 1993; (113): 345–361. 10.1037/0033-2909.113.2.345 8451339

[56] Somfai L. Béla Bártok: Composition, Concepts, and Autograph Sources Berkeley: University of California Press; 1996.

[57] Taubman H. The maestro, the life of Arturo Toscanini New York: Simon and Schuster; 1951.

[58] Collier GL, Collier JL. Studies of tempo using a double timing paradigm. Music Perception. 2007; 24(3): 229–245.

[59] Boltz MG. Changes in internal tempo and effects on the learning and remembering of event duration. Journal of Experimental Psychology: Learning, Memory, and Cognition. 1994; 20(5): 1154–1171.

[60] Kuhn TL. Effects of dynamics, halves of exercises, and trial sequences on tempo accuracy. Journal of Research in Music Education. 1977; 25(3): 222–227.

[61] Madsen CK. Modulated beat discrimination among musicians and non musicians. Journal of Research in Music Education. 1979; 27(2): 55–67.

[62] Pauws S. Effects of song familiarity, singing training and recent song exposure on the singing of melodies. Proceedings of the Fourth International Conference on Music Information Retrieval ISMIR; 2003; Baltimora, USA. 2003. pp. 57–64.

[63] Lapidaki E. Temporal stability in repeated listening tasks In: Maroni M, Addessi AR, Caterina R, Costa M editors. Proceedings of the International Conference on Music Perception and Cognition; 2006; Bologna: Bononia University Press pp. 1140–1148.

[64] Lapidaki E. Stability of Tempo Perception in Music Listening. Music Education Research. 2000; 2(1): 25–44.

[65] Collier GL, Collier JL. An exploration of the use of tempo in Jazz. Music Perception. 1994; 1(3): 219–242.

[66] Fine P, Bull S. Memory for tactus and musical tempo: The effects of expertise and speed on keeping time. International Symposium on Performance Science; 2009.

[67] Bennet B, Murdock Jr. The serial position effect of free recall. Journal of Experimental Psychology. 1962; (5): 482

[68] Pierce JR. The Science of Musical Sound New York: Scientific American Books Inc.; 1983.

[69] Adelman JS, Stewart N. Absolute identification is surprisingly faster with more closely spaced stimuli In Sun R, Miyake N editors. Proceedings of the twenty-eighth annual conference of the Cognitive Science Society. Mahwah, NJ: Erlbaum; 2002 pp. 943–948.

[70] Drake C, Botte MC. Tempo sensitivity in auditory sequences: Evidence for a multiple-look model. Perception & Psychophysics. 1993; 54: 277–286. 8414886 10.3758/bf03205262

[71] Allen G. Speech rhythm: Its relations to performance universals and articulatory timing. Journal of Phonetics. 1975; 3: 75–86.

[72] Metronome by Ron. [Software application] Available: http://members.ozemail.com.au/ronfleckner/metronome/. Accessed 12 March 2012.

[73] Visual Music: Bach Concerto 3D [Audiovisual file]. Available: http://www.youtube.com/watch?v=AHVl79kqzkg. Accessed 18 September 2015.

[74] Audacity [Free computer software]. Available: http://audacity.sourceforge.net/. Accessed 13 June 2013.

[75] Terhardt E, Ward WD. Recognition of musical key: Exploratory study. Journal of Acoustic Society of America. 1982; 72(1): 26–33.

[76] Lockhead GR, Byrd R. Practically perfect pitch. Journal of Acoustical Society of America. 1981; 70: 387–389.

[77] Rakowski A, Morawska-Büngeler M. In search of the criteria for absolute pitch. Archive of Acoustics. 1987; 12: 75–87.

[78] Terhardt E, Seewan M. Aural key identification and its relationship to absolute pitch. Music Perception. 1983; 1: 63–83.

[79] Sadakata M, Desain P, Honing H. The Bayesian way to relate rhythm perception and production. Music Perception. 2006; 23(3): 269–288.

[80] Sternberg S, Knoll R. Perception, production, and imitation of time ratios by skilled musicians In Gibbon J, Allan L editors. Timing and time perception, Annals of the New York Academy of Sciences. New York: New York Academy of Sciences; 1984 423: 429–441. 10.1111/j.1749-6632.1984.tb23451.x 6588806

[81] Dowling WJ. The development of music perception and cognition In Deutsch D editor. The psychology of music New York: Academic Press; 1999 pp. 603–625.

[82] Peretz I. The nature of music from a biological perspective. Cognition. 2006; 100: 1–32. 16487953 10.1016/j.cognition.2005.11.004

[83] Peretz I, Coltheart M. Modularity of music processing. Nature Neuroscience. 2003; 6: 688–691. 12830160 10.1038/nn1083

[84] Trehub SE, Hannon EE. Infant music perception: Domain-general or domain-specific mechanisms? Cognition. 2006; 100(1): 73–99. 16380107 10.1016/j.cognition.2005.11.006

[85] Trainor LJ, Wu L, Tsang CD. Long-term memory for music: infants remember tempo and timbre. Developmental Science. 2004; 7(3): 289–296. 15595370 10.1111/j.1467-7687.2004.00348.x

[86] Bergeson TR, Trehub SE. Infants’ perception of rhythmic patterns. Music Perception. 2006; 23(4): 345–360.

[87] Stewart N, Brown GDA, Chater N. Absolute identification by relative judgment. Psychological Review. 2005; 112(4): 881–911. 16262472 10.1037/0033-295X.112.4.881

[88] Lacouture Y, Marley AAJ. Choice and response time processes in the identification and categorization of unidimensional stimuli. Perception and Psychophysics. 2004; 66(7): 1206–1226. 15751477 10.3758/bf03196847

[89] Durlach NI, Braida LD. Intensity perception. I. Preliminary theory of intensity resolution. Journal of the Acoustical Society of America. 1969; 46: 372–383. 5804107 10.1121/1.1911699

[90] Luce RD, Green DM, Weber DL. Attention bands in absolute identification. Perception & Psychophysics. 1976; 20: 49–54.

[91] Traisman M. The magical number seven and some other features of category scaling: Properties for a model of absolute judgment. Journal of mathematical Psychology. 1985; 29: 175–230.

[92] Kent C, Lamberts L. An exemplar account of the bow and set-size effects in absolute identification. Journal of Experimental Psychology: Learning, Memory, and Cognition. 2005; 31: 289–305. 15755246 10.1037/0278-7393.31.2.289

[93] Nosofsky RM. An exemplar-based random-walk model of speeded categorization and absolute judgment In Marley AAJ editor. Choice, decision, and measurement. Hillsdale NJ: Erlbaum; 1997 pp. 347–365.

[94] Petrov AA, Anderson JR. The dynamics of scaling: A memory-based anchor model of category rating and absolute identification. Psychological Review. 2005; 112: 383–416. 15783291 10.1037/0033-295X.112.2.383

[95] Karpiuk P, Lacouture Y, Marley AAJ. A limited capacity, wave equality, random walk model of absolute identification In Choice, decision, and measurement: Essays in honor of R. Duncan Luce. Mahwah NJ: Erlbaum; 1997 pp. 279–299.

[96] Marley AAJ, Cook VT. A fixed rehearsal capacity interpretation of limits on absolute identification performance. British Journal of Mathematical and Statistical Psychology. 1984; 37: 136–151.

[97] Stewart N. Absolute identification is relative: A replay to Brown, Marley, and Lacouture (2007) Psychological Review. 2007; 114(2): 533–538. 17500643 10.1037/0033-295X.114.2.533

[98] Zatorre RJ, Chen JL, Penhune VB. When the brain plays music: auditory-motor interactions in music perception and production. Nature Reviews Neuroscience. 2007; 8: 547–558 17585307 10.1038/nrn2152

[99] Todd NMA, Lee C. An auditory-motor model of beat Induction. International Computer Music Association. 1994; 88:89. Available: http://quod.lib.umich.edu/i/icmc/bbp2372.1994.023/—auditory-motor-model-of-beat-induction?rgn=main;view=fulltext Accessed 18 September 2015.

[100] Todd NM. The kinematics of musical expression. Journal of Acoustical Society of America. 1995; 97: 1940–1949.

[101] Finney SA, Palmer C. 2003. Auditory feedback and memory for music performance: Sound evidence for an encoding effect. Memory & Cognition. 2003; 31: 51–64. 12699143 10.3758/bf03196082

[102] Palmer C, Krumhansl CL. Mental representations for musical meter. Journal of Experimental Psychology: Human Perception and Performance. 1990; 16(4): 728–741. 2148588 10.1037//0096-1523.16.4.728

[103] Patel AD. Language, music, syntax and the brain. Nature Neurosicence. 2003; (6): 674–681. 10.1038/nn1082 12830158

[104] Koelsch S, Gunter T, Wittfoth M, Sammler D. Interaction between Syntax Processing in Language and in Music: An ERP Study. Journal of Cognitive Neuroscience. 2005; 17(10): 1565–1577. 16269097 10.1162/089892905774597290

[105] McFarland DJ, Cacace AT. Aspects of short-term acoustic recognition memory: Modality and serial position effects. Audiology. 1992; 31, 342–352 1492818 10.3109/00206099209072922

[106] Parmentier FBR, Mayberry MT, Jones DM. Temporal grouping in auditory spatial serial memory. Psychonomic Bulletin & Review. 2004; 11: 501–507. 15376802 10.3758/bf03196602

